# A Review of Biomonitoring of Phthalate Exposures

**DOI:** 10.3390/toxics7020021

**Published:** 2019-04-05

**Authors:** Yu Wang, Hongkai Zhu, Kurunthachalam Kannan

**Affiliations:** 1 Wadsworth Center, New York State Department of Health, Albany, NY 12201, USA; wangyu@mail.nankai.edu.cn (Y.W.); Hongkai.Zhu@health.ny.gov (H.Z.); 2 Department of Environmental Health Sciences, School of Public Health, State University of New York at Albany, Albany, New York, NY 12201, USA

**Keywords:** phthalate, DEHP, biomonitoring, human exposure, toxicity, reproductive

## Abstract

Phthalates (diesters of phthalic acid) are widely used as plasticizers and additives in many consumer products. Laboratory animal studies have reported the endocrine-disrupting and reproductive effects of phthalates, and human exposure to this class of chemicals is a concern. Several phthalates have been recognized as substances of high concern. Human exposure to phthalates occurs mainly via dietary sources, dermal absorption, and air inhalation. Phthalates are excreted as conjugated monoesters in urine, and some phthalates, such as di-2-ethylhexyl phthalate (DEHP), undergo secondary metabolism, including oxidative transformation, prior to urinary excretion. The occurrence of phthalates and their metabolites in urine, serum, breast milk, and semen has been widely reported. Urine has been the preferred matrix in human biomonitoring studies, and concentrations on the order of several tens to hundreds of nanograms per milliliter have been reported for several phthalate metabolites. Metabolites of diethyl phthalate (DEP), dibutyl- (DBP) and diisobutyl- (DiBP) phthalates, and DEHP were the most abundant compounds measured in urine. Temporal trends in phthalate exposures varied among countries. In the United States (US), DEHP exposure has declined since 2005, whereas DiNP exposure has increased. In China, DEHP exposure has increased since 2000. For many phthalates, exposures in children are higher than those in adults. Human epidemiological studies have shown a significant association between phthalate exposures and adverse reproductive outcomes in women and men, type II diabetes and insulin resistance, overweight/obesity, allergy, and asthma. This review compiles biomonitoring studies of phthalates and exposure doses to assess health risks from phthalate exposures in populations across the globe.

## 1. Introduction

**Figure 1:**
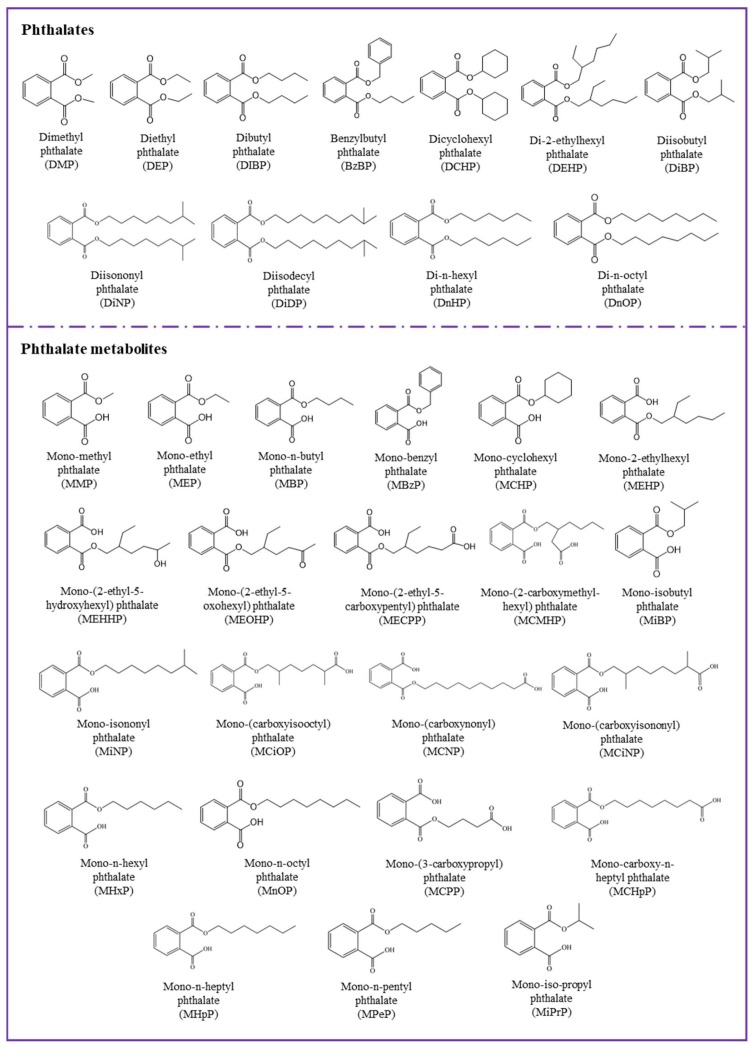
Chemical structures of major phthalates and their metabolites studied in the literature.

Phthalates are diesters of phthalic acid (1,2-benzenedicarboxylic acid) and are synthetic organic chemicals used in industries as solvents, plasticizers, and additives in polyvinyl chloride (PVC) plastics or personal care products (PCPs) [1]. More than 25 phthalates are used in commercial applications, with each adding unique qualities to the product into which it is incorporated. Ten commonly used phthalates (Figure 1, Table 1) are dimethyl phthalate (DMP), diethyl phthalate (DEP), dibutyl phthalate (DBP), diisobutyl phthalate (DiBP), benzylbutyl phthalate (BzBP), dicyclohexyl phthalate (DCHP), di(2-ethylhexyl) phthalate (DEHP), di-n-octyl phthalate (DnOP), di-isononyl phthalate (DiNP), and di-isodecyl phthalate (DiDP). DEHP, one of the major phthalates in commerce, was first synthesized for use as a plasticizer in 1933 [2]. The application of DEHP as an additive in polyvinyl chloride (PVC) to impart the flexibility of plastic has made phthalates popular around the world. The addition of phthalates to PVC makes it not only flexible but also malleable and durable. PVC products may contain up to 50% (by weight) phthalates [1].

Currently, phthalates are used in many types of products, including building materials, automotive parts, medical devices, food packaging, cosmetics, perfumes, toys, teethers, adhesives, paints, floorings, lubricants, hair sprays, shampoos, soaps, nail polishes, and detergents [3,4,5]. The annual global production of phthalate was 4.7 million metric tons in 2006 [6,7] and ~8 million metric tons in 2015 [8]. In most commercial products, DEHP, DiNP, and BzBP are used as additives, and they easily migrate from those products into the environment through evaporation, leaching, and abrasion [9]. Phthalates have been measured in a range of environmental matrices, including sludge, dust, soil, air, and water [4], and are ubiquitous contaminants in the environment.

Phthalates are reproductive and developmental toxicants [10]. In laboratory animal studies, DEHP has been reported to affect the reproductive system and development [11,12]. Further, changes in hepatic structure and function and kidney function as well as disruption of thyroid signaling, immune function, and metabolic homeostasis were reported [13,14,15,16]. The US Environmental Protection Agency (EPA) classified DEHP and BzBP as probable and possible human carcinogens, respectively. European authorities have classified phthalates with three to six carbons in their backbone as Repr 1B Agents (i.e., presumed human reproductive toxicants) (https://echa.europa.eu/substance-information/-/substanceinfo/100.239.213).

Human exposure to phthalates arises mainly from ingestion, inhalation, and dermal absorption [17,18]. Human biomonitoring studies have measured parent phthalate in serum [19] and their metabolites in human urine [20,21], semen [22,23], and breast milk [24,25]. Studies have demonstrated that phthalate exposure is associated with oxidative stress in humans [26,27]. Some studies have linked phthalate exposure to premature thelarche [28,29], endometriosis [30,31], low semen quality [32], reduced testosterone levels [33], obesity, diabetes, and breast cancer [34,35]. Phthalates are regarded as endocrine-disrupting compounds [36]. One of the most significant effects of phthalates is in terms of fetal development and reproductive anomalies and is referred to as “phthalate syndrome” (e.g., developmental or testicular effects, insulin like factor 3 production) [37,38]. In addition, phthalate exposure might be linked to insulin resistance and obesity in human populations [39,40].

In 1999, the European Union (EU) temporarily banned the use of six phthalates in children’s toys: DiNP, DEHP, DBP, BzBP, DiDP, and DnOP (http://europa.eu/rapid/press-release_IP-05-838_en.htm). Further, in 2009, these phthalates were restricted in toys in Europe (https://eur-lex.europa.eu/legal-content/EN/TXT/?uri=CELEX%3A32009L0048). The US followed suit in 2008 by passing the Consumer Products Safety Improvement Act, which banned the same six phthalates in children’s toys (https://www.cpsc.gov/Regulations-Laws--Standards/Statutes/The-Consumer-Product-Safety-Improvement-Act). Many industries began substituting alternative chemicals for phthalates in their products, and several substitutive phthalate and non-phthalate plasticizers are currently used in many products [41,42]. Although six phthalates are now restricted in children’s products in the US and EU, they are unregulated and continue to be used in toys in many other parts of the world, including China and India. In addition, children continue to be exposed to phthalates in cosmetics and PCPs as well as in school supplies made of PVC, including notebooks and binders, art supplies, backpacks, lunchboxes, paperclips, and umbrellas (https://www.sustainableproduction.org/downloads/PhthalateAlternatives-January2011.pdf). Raincoats, boots, handbags, and soft plastic shoes also may contain phthalates.

A search on the basis of Web of Science Core Collection, BIOSIS Previews, Derwent Innovations Index, MEDLINE, and ScieELO Citation Index was carried out to identify studies relevant to biomonitoring and epidemiology on phthalates and phthalate metabolites. Topics of interest included studies on phthalates in urine, serum, and other biofluids. The search terms used were: phthalic acid/phthalates OR phthalate metabolites AND biomonitoring OR epidemiological studies. Publications between 2000 and 2018 were extracted. This review provides a summary of human biomonitoring studies of phthalate diesters and their monoester (primary) and oxidative (secondary) metabolites as well as select epidemiological studies that link phthalate exposure to health outcomes in human populations.

## 2. Sources of Phthalates

Owing to their widespread use in consumer products, phthalates are ubiquitous in the environment, and a variety of sources have been reported to contribute to human exposure. For the purpose of exposure analysis, phthalates have often been grouped as lower molecular weight (ester side-chain lengths, one to four carbons; DMP, DEP, and DBP), and higher molecular weight (ester side-chain lengths, five or more carbons; DEHP, DiNP, DiDP, and BzBP) phthalates [43]. The high molecular weight phthalates are used primarily in PVC polymers and plastisol applications, plastics, food packaging, and food processing materials, vinyl toys and vinyl floor coverings, and building products. The low molecular weight phthalates are often used in non-PVC applications, such as personal care products, paints, adhesives, and enteric-coated tablets [44]. BzBP, DEHP, DiNP, DBP, and DiBP are used in toys, bags, gloves, and plastic tubing for improving flexibility and making the polymeric products soft and malleable [4]. DMP and DEP are widely used in cosmetics, such as perfumes, aftershaves, shampoos, makeup, and nail care products [4]. Cosmetics and personal care products are the major sources of human exposure to low molecular weight phthalates. Food packaging plastic film contains phthalates (such as DBP and DEP) at levels of up to 10% by weight. Plasticizer migration occurs when food packaging films come in direct contact with foods, and fatty foods and high temperatures increase the migration [45]. Diet has been a major source of exposure to high molecular weight phthalates, especially DEHP. In particular, foods packaged in plastic/PVC materials contribute to exposure to DEHP in humans [46].

The major source of exposure to DEP—one of the major phthalates found in human urine—is cosmetics and personal care products [17]. Studies have reported elevated concentrations of phthalates in indoor air and dust [47]. In fact, among various contaminants measured in indoor dust, phthalates, especially DEHP and DEP, are the major contaminants in indoor dust and air [46]. Phthalates also were reported to occur in pharmaceuticals, especially in over-the-counter medications/syrups and in pills with enteric coatings [48,49]. Medical devices that are suspected to contain DEHP include intravenous (IV) storage bags, ventilator tubing, IV infusion sets, endotracheal tubes, IV infusion catheters, nasogastric tubes, blood storage bags, enteral and parenteral nutrition storage bags, blood administration sets, urinary catheters, PVC exam gloves, suction catheters, chest tubes, nasal cannula tubing, hemodialysis tubing, syringes, extracorporeal membrane oxygenation tubing, and cardiopulmonary bypass tubing [50].

Exposure doses to phthalates have been calculated through the ingestion of foods, air inhalation, and dust ingestion for the general population in the US (sampled during 2011–2014) (Table 2) [46]. Dust ingestion is a major source of exposure to phthalates in infants and toddlers, whereas diet is the major source for children and adults. The exposure doses are in the range of a sub to low µg/kg bw/d. Further details of exposure doses calculated through biomonitoring data are provided below.

## 3. Biomonitoring of Phthalates

Due to the ubiquitous occurrence and widespread exposure of phthalates, their metabolites are one of the most examined environmental chemicals in human biomonitoring studies. The reported half-life of phthalates diesters in blood plasma or urine of humans and rodents was less than 24 h. Several studies have reviewed pharmacokinetics of phthalate esters, and these studies have found rapid hydrolysis of diesters to monoesters in the gastrointestinal tract [1,2]. Binding of DEHP metabolites to blood plasma proteins, existence of biliary excretion, and enterohepatic circulation in humans have been suggested [2]. Nevertheless, urinary excretion has been the major elimination pathway of phthalates [2]. Urinary concentrations of phthalate metabolites are generally 5–20 times higher than that in lipid-rich compartments. For example, urinary concentrations of mono-2-ethylhexyl phthalate (MEHP), mono-isobutyl phthalate (MIBP), mono-ethyl phthalate (MEP), and mono-n-butyl phthalate (MBP) were 20–100 times those in blood or milk [24]. Phthalate metabolites have been measured in various body fluids, including urine [47,51], serum [52,53], semen [32,54], breast milk [55,56], and saliva [57] (Table 3). Phthalates can cross the placental barrier [58] and have been measured in amniotic fluid in human studies [59]. To date, studies that report partitioning of phthalates among various tissues and organs in an organism, at state-state exposure conditions, are not available. It is worth noting that a few earlier reviews have described biomonitoring of phthalates in humans [60]. Biomonitoring studies that report concentrations of phthalates metabolites are presented in Table 3.

Phthalate diesters (parent compounds) were measured in blood plasma of women with endometriosis in India, and a significant association was found between phthalate exposure and the risk of developing endometriosis [136]. Similarly, studies have determined phthalates in serum samples of couples from Greenland, Poland, and Ukraine that showed that the DEHP levels were associated with reduced time to achieve pregnancy [137]. Phthalate diesters and their metabolites also have been measured in breast milk, serum, and urine from Swedish women [24]. In milk and serum samples, the concentrations of phthalate diesters and their metabolites were below the method limit of detection (0.12–3.0 µg/L). Detectable concentrations of phthalate metabolites, however, were found in urine (0.1–1000 µg/L). Measurements of phthalate diesters in breast milk and serum are prone to false positives due to background contamination. Medical devices, including blood collection devices and plastic containers that are used to collect and store samples, can contain phthalate diesters [49]. If the samples were to be analyzed for phthalate diesters, caution should be taken with the screening devices used to collect and store samples. A comprehensive review of challenges associated with low-level phthalate analysis in biological specimens has been published [17].

### 3.1. Phthalate Metabolites in Urine

**Figure 2:**
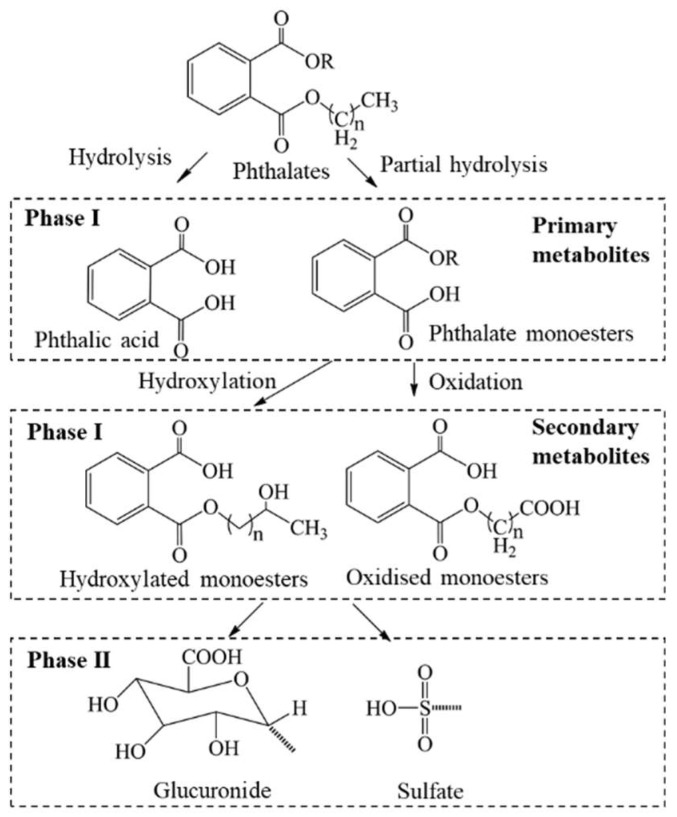
Metabolic pathways of phthalate esters in humans.

Although microbial degradation of DEHP to MEHP in soils through lipase and esterase enzymes has been shown, environmental degradation/transformation of parent phthalates is slow [25,138]. Because phthalates have a short half-life in human bodies and are excreted quickly in urine as monoester metabolites, the metabolites are suitable biomarkers for human exposure to parent compounds. The half-life of phthalates in human bodies (in plasma and urine) is less than 24 h, and following metabolism, monoesters of phthalates are conjugated with glucuronide or sulfate and excreted in urine [139]. Analysis of metabolites in urine involves enzymatic deconjugation followed by purification. Assessment of human exposure to phthalates is based mainly on the measurement of their urinary monoester metabolites, although several secondary and oxidative metabolites have been reported to occur in human specimens [139]. For instance, DMP, DEP, and DBP undergo degradation/hydrolysis and form their corresponding monoesters, i.e., MMP, MEP and MBP, respectively. Both hydrolysis and oxidation products are formed from the metabolism of DEHP. MEHP, the hydrolysis product of DEHP, is not a major metabolite. The oxidative metabolites, MEOHP, MEHHP, MECPP, and MCMHP, however, are the major metabolites of DEHP and are appropriate biomarkers of exposure to this compound [21]. Some studies suggest, however, that MEHP is more toxic than are other oxidative metabolites. The general metabolic pathways of phthalate esters in humans are shown in Figure 2.

General Population Adults: A large number of studies have reported measurements of phthalate metabolites in human specimens collected from European (Germany, Netherlands, Denmark, Norway, Sweden, Greece, the Czech Republic, Hungary, Slovakia, and Spain) and Asian countries (Japan, China, South Korea, India, Taiwan, Vietnam, Saudi Arabia, Malaysia, and Kuwait) as well as from North American countries. The number of phthalate metabolites measured in urine samples varied considerably; as new analytical standards become made available commercially, more metabolites were added to the list of compounds measured in urine. Although a majority of the recent studies measure close to 20 phthalate metabolites, studies conducted a decade ago measured 10 or fewer metabolites of phthalates.

In general, the concentrations of the sum of 22 phthalate metabolites measured in human urine were on the order of several to hundreds of parts-per-billion (µg/L) [21]. In a majority of the biomonitoring studies, metabolites of DEHP, DEP, and DBP were the major compounds identified in urine, and the profile varied depending on the country. Urine samples collected from 32 men and 53 women (age: 7–64 years) from northern Bavaria (Germany) contained MBP (median: 181 µg/L), MEP (90.2 µg/L), and major DEHP metabolites, such as MEHHP (46.8 µg/L) and MEOHP (36.5 µg/L) [86]. The median concentrations of DEHP metabolites, namely, MEHP, MEOHP, and MEHHP, were 4.5, 28.3, and 35.9 µg/L, respectively, and these three metabolites were highly intercorrelated. The concentration ratios, MEHHP/MEHP, and MEOHP/MEHP, were calculated to be 8.2, and 5.9, respectively. These ratios suggest that MEHP is further oxidized to form MEHHP and MEOHP [86].

The urinary concentrations of phthalate metabolites in general populations vary among countries. Some of the highest concentrations of total phthalate metabolites were found in urine collected in 2006–2007 from Kuwaitis, with a maximum value of 19,300 µg/L and a median value for of 1050 µg/L [72]. The occurrence of phthalate metabolites was investigated in urine from Germans, and MBP was found at high concentrations (median: 49 µg/L) [87]. The median concentrations of phthalate metabolites in urine samples from Germany decreased significantly from 2002 to 2008 [41]. Similarly, urinary phthalate metabolites measured in 2015 were significantly lower than those in 2007 in Germany [88].

Biomonitoring studies in other European countries, including France [83], Belgium [65,66], Slovakia [117], and Norway [112], report phthalate metabolite concentrations in the range of 1–100 μg/L in urine from adults. MBP and DEHP metabolites were the predominant compounds found in those studies. Further, a comparative analysis of biomonitoring data in Europe suggested a significant decline in phthalate metabolite concentrations (especially MEP, MBP, MBzP, and DEHP metabolites) from 2011 to 2016 [42]. Several alternative plasticizers, however, are used as replacements for DEHP in European countries. Common alternatives include Hexamoll DINCH (DINCH), acetyl tributyl citrate (ATBC), dioctyl terephthalate (DOTP), 2,2,4-trimethyl 1,3-pentanediol diisobutyrate (TXIB), trioctyl trimellitate (TOTM), and di-(2-ethylhexyl) adipate (DEHA).

In North America, the distribution of phthalate metabolites in urine has been summarized in nationwide monitoring surveys. For example, the US National Health and Nutrition Examination Survey (NHANES) of the Centers for Disease Control and Prevention (CDC) showed that MEP, MEHP, MEHHP, and MEOHP concentrations in urine from adults >20 years of age were 167, 3.99, 18.8, and 12.6 µg/g creatinine (CR), respectively [129]. The NHANES program has measured 15 phthalate metabolites in urine. The weighted geometric mean concentration of 15 phthalate metabolites in the US general population was 125 µg/L for the samples collected in the period of 2007–2008. MEP was the major compound found in urine, accounting for >70% of the total concentrations, which was followed by mono-(2-ethyl-5-hydroxyhexyl) phthalate (MEHHP; ~18% of the total phthalate concentrations). The NHANES data for the US general population in the period of 2005–2006 showed that MCNP, a metabolite of DiDP, was found at a median concentration of 2.70 µg/L [140]. The updated NHANES report for 2013–2014 are available (https://wwwn.cdc.gov/Nchs/Nhanes/2013-2014/SSPHTE_H.htm). A 67% decline in DEHP exposure in the US population between 2005/6 and 2011/12 has been reported [141]. Several factors have been shown to affect exposures. The NHANES data showed that several phthalate urinary metabolites were higher in males, Hispanics, and African Americans [142]. The Human Biomonitoring Program of Health Canada measured 11 phthalate metabolites in urine samples of 3236 Canadians and found median MEP and MEHHP concentrations at 49.1 and 23.4 µg/L, respectively [68]. Since 2001, there has been clear evidence of a decline in DEP, DBP, and DEHP exposure in the US [115]. In contrast, urinary DiNP concentrations in the US population increased significantly during the period 2005/6–2011/12 (www.cdc.gov/exposurereport).

Urinary concentrations of phthalate metabolites have been reported for several Asian countries [97]. The measured concentrations in Asian countries were similar to those reported in Europe and North America, although the profiles were distinct. For instance, MBP and MiBP were the major metabolites found in urine from China, and their respective median concentrations were 61.2 and 51.7 μg/L [51]. Similar concentrations of MBP and the sum of DEHP metabolites were reported in urine from Nanjing city (47.1 and 42.0 μg/L) [74] and Taiwan (47.1 and 42.0 μg/L) [121]. In contrast, DEHP metabolites were predominant in urine from Japan, Malaysia, and Vietnam [72]. A nationwide survey of urine samples from 6478 adults during the period of 2012–2014 in Korea showed median urinary concentrations of DEHP metabolites (88.2 μg/L) that were twofold higher than that of MBP (44.2 μg/L) [106,107]. In Israel, phthalate metabolites were found in urine samples collected from 250 adults (ages 20−74), with median concentrations that ranged from 17.1 μg/L (MEOHP) to 37.6 μg/L (MiBP) [97]. DEHP exposure in the Chinese population has increased since 2001 [143].

**Figure 3:**
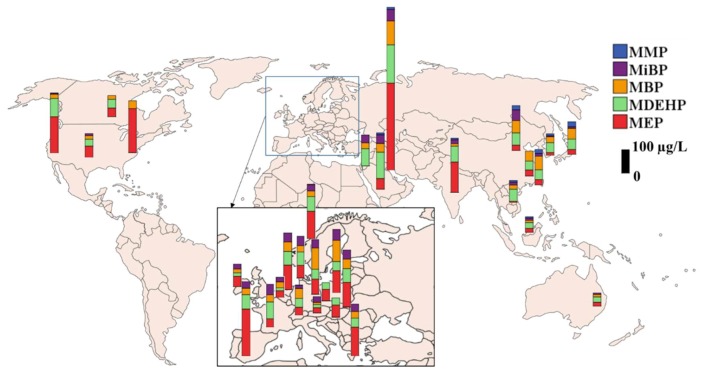
Urinary concentrations of phthalate metabolites reported in adults (general population) from several countries (MDEHP: Sum of DEHP metabolites; biomonitoring data published after 2000; median concentration is presented).

The global distribution of major phthalate metabolites measured in urine from general populations is presented in Figure 3. Urine samples collected from Kuwait during 2006-2007 contained the highest median concentrations of MEP (411 μg/L), MBP (113 μg/L), and DEHP metabolites (180 μg/L), with a sum of median phthalate metabolite concentrations (median) at 1,050 μg/L [72]. This value is the highest among all countries studied. The profiles of phthalate metabolites varied, with MEP as the predominant metabolite in Indian and Kuwaiti urine samples (49% of the total), which were similar to those found in the US. In China (52%), MBP was the major metabolite found in urine. In Korea (46%), Japan (31%), and Vietnam (52%), DEHP metabolites were the dominant ones. MMP accounted for <8% of the total phthalate metabolite concentrations in all Asian countries, except for Japan, where it was 20%. Overall, MEP and DEHP metabolites were the major phthalate metabolites found in urine from most Asian countries, a pattern similar to that found in the US [130]. The reported urinary concentrations of phthalate metabolites among several European countries were similar whereas information for African countries and Australia/Oceanian countries is limited.

Pregnant Women: Phthalates have been widely studied for exposure levels in pregnant women. MEP (222 µg/g CR) was the predominant phthalate metabolite found in urine samples of pregnant women from the Netherlands (Generation R study) [110]. Similar exposure levels were reported for pregnant women from the US [131,132], Canada [69], and Norway [113], with MEP median concentrations exceeding 30 µg/L. In a study of urinary phthalate metabolite concentrations in Spanish pregnant women (*n* = 391), the median concentration of MEP was reported at 246 μg/g CR [118].

Several studies have examined phthalate metabolite concentrations in matched urine samples of newborns and mothers. Maternal urinary concentrations of MEHHP and MEOHP in Korea were 17.7 and 14.7 µg/L, respectively, which were two- to threefold higher than those found in newborns (5.79 and 3.27 µg/L) [144]. Another study, however, showed similar urinary concentrations of phthalate metabolites between 120 mother-and-child pairs [96]. Occurrence of phthalate metabolites in pregnant women suggests potential exposure in the fetus.

**Figure 4:**
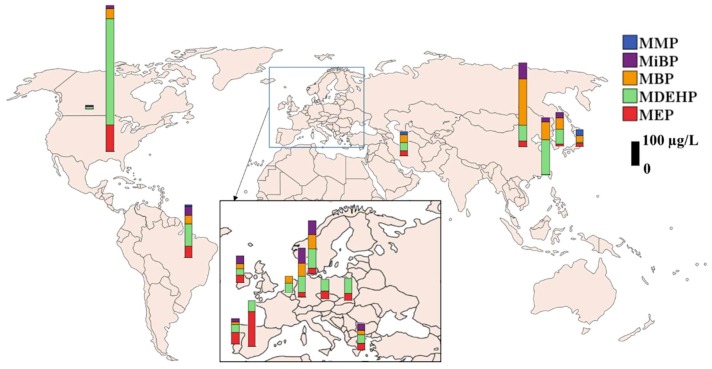
Urinary concentrations of phthalate metabolites reported in children (general population) from several countries (MDEHP: Sum of DEHP metabolites; biomonitoring data published after 2000, median concentration is presented).

Children: The NHANES data showed that the concentrations of urinary phthalate metabolites in children 6–11 years old were higher than those in adolescents and adults [142]. Several studies support the CDC’s findings that children have higher urinary concentrations than do adults of DBP, BzBP, and DEHP [41,145]. Differences in urinary concentrations of phthalates among infants, children, and adults may reflect different sources and routes of intake. Ingestion is thought to be a primary pathway of exposure to some phthalates, especially those in food packaging [146]. The mouthing behavior of infants and toddlers could potentially increase their exposures to phthalates in toys and other products made with plasticized polymers. The global distribution of reported urinary phthalate metabolite concentrations in children is shown in Figure 4.

MEP, MBP, and DEHP metabolites were the dominant compounds detected in urine from children. Spot urine samples from 5- to 7-year-old German children contained a median phthalate metabolite concentration (sum of 5 metabolites) of 76.9 µg/L, with DEHP metabolites as major compounds [89]. A similar concentration of DEHP metabolites at 75.7 µg/L was found in urine samples from 8- to 10-year-old German children (*n* = 465) [90]. Several biomonitoring studies reported comparable concentrations of DEHP metabolites and MBP in urine from children in China [75], Korea [108], Canada [70], Brazil [67] and Portugal [114].

In urine samples collected from children in Beijing, China, MBP was the most abundant metabolite (median: 232 µg/L), followed by MiBP (81.3 µg/L), MECPP (79.1 µg/L), and MEP (28.5 µg/L). A significant association between the concentrations of parent phthalate diesters in handwipes and the corresponding monoester metabolites in urine were observed in urine from children, which suggested that dermal absorption is an important exposure pathway for phthalates in children [75]. Mean urinary concentrations of MBP decreased as the children aged [91]. Among children, urinary DBP and DEHP metabolites in boys were higher than those in girls, whereas urinary MEP concentrations were positively correlated with age in both genders [79]. Urinary concentrations of MEP in adolescents were higher than those in children, which was associated with high cosmetic usage among teenagers [79,95].

Urinary phthalate metabolite concentrations have been reported for children and adults from 17 European countries, namely, Belgium, Cyprus, Czech Republic, Denmark, Germany, Hungary, Ireland, Luxembourg, Poland, Portugal, Romania, Slovenia, Slovak Republic, Spain, Sweden, Switzerland, and the United Kingdom (DEMOCOPHES); the geometric mean concentrations of MEP, MBZP, MBP, MiBP, and ΣDEHP metabolites were 34.4, 7.15, 34.8, 45.4, and 47.6 µg/L for children (*n* = 1355) and were 48.2, 4.51, 23.9, 30.1, and 29.2 µg/L for mothers [81], which suggested that children in those countries were more highly exposed to several phthalates than were their mothers. Nevertheless, some studies reported higher urinary MEP concentrations in mothers (45.1–72.0 μg/L) than in children (12.1–16.4 μg/L) [82,92]. The concentrations of DEHP metabolites were reported to be similar between mothers and children [82,109,119]. A significant positive correlation existed in urinary phthalate metabolite concentrations between children and their parents. MECPP, an oxidative metabolite of DEHP, was predominant in urine from children (92.7%) relative to that found in adults (56.7–57.6%). Studies have found that children possess enhanced oxidative metabolism for DEHP [91,109,147]. Another study of urinary phthalate metabolites in 104 paired mothers and school-aged children reported higher concentrations of secondary DEHP metabolites in children than in mothers [93]. A study from Austria showed higher urinary concentrations of phthalate metabolites in children than adults [62]. Overall, these studies suggest higher exposures to phthalates in children than adults.

Highly Exposed Populations: Highly exposed individuals have urinary phthalate metabolite concentrations that often exceed those at the 95th percentile of the general population (https://www.ncbi.nlm.nih.gov/books/NBK215044/). Neonates who receive medical treatments such as transfusions are widely recognized as potentially highlexposed [148]. A study from Slovakia showed that the urinary concentrations of DEHP metabolites, MiBP, and MBP in occupationally exposed individuals from plastic industry were 55.9, 39.2, and 110 µg/L, respectively [115], which were higher than those in urine from women of no known occupational exposures [61]. The median concentrations of MEP, MBP, MiBP, and DEHP metabolites in urine from hairdressing apprentices who attended vocational training schools in Slovakia were 201, 103, 61.4, and 82.7 µg/L, respectively [116]. Some medications contain phthalates in their coatings or delivery systems [49] and may contribute to the high exposures of children, pregnant women, and others who take these medications.

Exposure Assessment: The concentrations of phthalate metabolites measured in urine can be used to assess the amount of parent phthalate to which humans are exposed, when the fraction of the metabolite excreted in urine is known, as presented in the equation below [147]:

(1) $$\mathrm{Estimated}\;\mathrm{parent}\;\mathrm{phthalate}\;\mathrm{concentration}=\frac{\mathrm{Metabolite}\;\mathrm{concentration}}{\mathrm{Excretion}\;\mathrm{fraction}}$$

The estimated daily intake (*EDI*) of parent phthalates is then calculated by taking the average weight of an individual with the average urinary excretion rate, as shown in the equation below:

(2) $$\begin{array}{l} \mathrm{Estimated}\;\mathrm{daily}\;\mathrm{intake}\;\left(\mathrm{EDI}\right)\\ =\frac{\mathrm{Estimated}\;\mathrm{parent}\;\mathrm{phthalate}\;\mathrm{concentration}\;\times \mathrm{Daily}\;\mathrm{urine}\;\mathrm{excretion}\;\mathrm{volume}\;}{\mathrm{Average}\;\mathrm{body}\;\mathrm{weight}} \end{array}$$

Several studies have estimated exposure doses to phthalates in populations, which allowed for comparison against a reference dose (*RfD*), the maximum acceptable oral dose of a toxic substance, of the US EPA. The estimated mean daily exposure doses to DEP and DBP in Asian countries and the US were one to two orders of magnitude below the EPA *RfD* (DEP = 800, DBP = 100, and DEHP = 20 µg/kg body weight (bw)/day). The estimated daily exposure doses to DEHP in Kuwait and India, however, were close to the RfD of the US EPA [72]. Similarly, high concentrations of DEHP metabolites (mean concentration = 338 μg/L) were reported in urine from the Saudi population [26].

The calculated *EDI*_max_ values for DEHP and DBP were 8 and 0.08 µg/kg bw/day, respectively, for the population in Taiwan, which were one to two orders of magnitude lower than the tolerable daily intake (*TDI)* values (the daily intake amount of a chemical that has been assessed to be safe for human being on a long-term basis) suggested for DEHP (50 µg/kg bw) and DBP (10 µg/kg bw) by the European Food Safety Authority (EFSA) [147].

The 95th percentile for DEHP exposure doses calculated for the general population (*n* = 85) and children (*n* = 254) from Germany were 21 and 25 µg/kg bw/day, respectively, which exceeded the RfD (20 µg/kg bw/day) and the TDI (20–48 µg/kg bw/day) [149]. Further, elevated exposure to phthalates, especially DEHP, in neonates admitted to intensive care units was reported (median: 42 µg/kg bw/day; 95th percentile: 1780 µg/kg bw/day) [149], and the exposure dose was higher than the RfD.

Some studies defined “Biomonitoring Equivalents (BEs)” as the concentration or range of concentrations of a chemical or its metabolite in a biological medium (blood, urine, or other medium) that is consistent with an existing health-based exposure guideline (e.g., RfD and TDI) [150,151]. BE values for MBP, MBzP, and MEP were reported at 18000, 3800 and 2700 µg/L, respectively [150], and the BE values range from 1500 to 3600 µg/L for MiNP [151]. These values may be used as screening tools for evaluation of biomonitoring data for phthalate metabolites in the context of existing risk assessments and for prioritization of the potential need for additional risk assessment efforts for each of these compounds relative to other chemicals [150,151].

Although current exposure doses in the general population are below the tolerance limits reported by environmental agencies, certainly population groups, especially children, are exposed to high levels of phthalates. Studies of the effects of phthalates from early life stage exposures are warranted.

### 3.2. Phthalate Metabolites in Serum

The biomonitoring studies of human phthalate exposure have been based on urinary concentrations of phthalate metabolites. However, when only serum was available for analysis, MEP and MiBP representing low molecular weight phthalates, and MECPP and MCiOP representing high molecular weight phthalates, have been used as indicators of phthalate exposure [77]. A study reported the correlations of phthalate metabolite concentrations among urine, serum, and seminal plasma of young Danish men [77]. The mean concentrations of MEP, MBP, and DEHP metabolites were one to two orders of magnitude lower in serum (MEP: 4.2, MBP: 0.4, and DEHP: 7.6 µg/L) and seminal plasma (1.0, 0.8, and 0.6 µg/L) than in urine (326, 42.5, and 115 µg/L). Another study, however, showed that the distribution pattern of monoester metabolites in serum was similar to that of urine [152], especially for MEHP (the metabolite of DEHP) [152]. Nevertheless, MEHHP, MEOHP, MECPP, and MCMHP were found at much higher concentrations in urine than in serum [153]. The presence of MEHP in serum was more likely related to contamination that arises from sampling devices.

Whole blood and cord blood samples from 128 healthy pregnant women and their newborns were analyzed for phthalate metabolites. Median concentrations of MEHHP and MEOHP were 0.31 and <LOD µg/L in maternal blood and 0.32 and <LOD µg/L in cord blood, respectively. MEHHP and MEOHP also were reported to occur in the placenta at concentrations of 0.09 and <LOD ng/g [144]. MBP, MEHP, MEP, and MiBP were detected in blood serum at median concentrations of 0.54 0.49, 0.50, and 0.5 µg/L, respectively [24], and these concentrations were at least an order of magnitude lower than those measured in urine.

In the serum of patients who were undergoing dialysis [53,154,155,156], phthalate acid (PA) was found as a metabolite of phthalates at remarkably high concentrations of 5.22 ± 3.94 mg/L [155]. Another study also reported the occurrence of PA in serum (0.205 ± 0.067 mg/L) of patients who were undergoing dialysis [154]. Accumulation of PA in patients who are undergoing dialysis has been suggested [156]. Serum concentrations of MEHP and DEHP were reported in autistic children [157].

### 3.3. Phthalate Metabolites in Amniotic Fluid, Breast Milk, Semen, and Saliva

MBP was found in >93% of amniotic fluid samples collected from the US [59] at concentrations two- to threefold lower than those of serum and four- to sevenfold lower than those of urine [59]. Studies have reported the occurrence of phthalates in breast milk [158]; the reported concentrations in breast milk were much lower than those in urine but similar to those in amniotic fluid. Monoester metabolites of phthalates were measured in breast milk from 33 lactating mothers in North Carolina. MCPP (0.2 µg/L) and MEOHP (0.3 µg/L), MECPP (0.1–0.4 µg/L), and MEHHP (0.2–0.3 µg/L) were detected in some samples [131]. MiNP was the major metabolite found in breast milk collected from mothers in Denmark (101 µg/L) and Finland (89 µg/L) [25,159]. Median concentrations of MBP, MBzP, and MEHP in breast milk were 0.54, 0.50, and 0.49 µg/L, respectively [24].

Human saliva samples (*n* = 39) also contained phthalate metabolites [57,160]. Salivary concentrations of phthalate metabolites in 39 adult volunteers were in the ranges of <1 to 10.6 µg/L for PA, 91.4 µg/L for MEP, 65.8 µg/L for MBP, and 354 µg/L for MBzP. MBP was the most (85%) frequently detected compound in saliva [57]. Two phthalate metabolites (2.2 µg/L MCPP and 2.3 µg/L MECPP) were detected in a saliva sample from a US woman [131].

MBP and MBzP were found in semen from US men [32,54]. High concentrations of DEHP and its metabolites (∑40.6 µg/L) were found in semen from German men [161]. Studies have also indicated that semen quality can be affected by environmentally relevant phthalate exposures [121]. Further, DEHP (4.20 µg/L) and DBP (2.06 µg/L) were reported at high concentrations in male seminal plasma from men in the US. The metabolites of DEHP (∑0.98 µg/L) and MBP (2.97 µg/L) also were present in considerable concentrations in seminal plasma in the same study [162]. These results suggested that phthalate metabolites can partition in seminal plasma. Similarly, DEHP (2.09 µg/L) and DBP (1.75 µg/L), as well as their metabolites, were found as the predominant phthalates/phthalate metabolites in seminal plasma from male partners who were planning for pregnancy. This study showed adverse associations between seminal phthalate metabolite concentrations and semen quality [163].

Phthalate metabolites were measured in nail samples from Belgium, and the total concentrations ranged between <12 and 7980 ng/g. It should be noted, however, that some phthalates, especially DBP, are used in nail polishes and that care should be exercised in interpreting such measurements. MEHP, MBP, and MEP were the major metabolites detected in every nail sample, with a median concentration of 138, 74, and 64 ng/g, respectively [135]. Another study of nail samples from Oslo, Norway, showed the presence of monoesters, such as MMP (geometric mean 89.7 ng/g), MEP (104.8 ng/g), and MBP (89.3 ng/g) [112]. The utility of other biologic matrices, such as blood, breast milk, semen, and nails, for assessing human exposure to phthalates remains largely unknown due to the limited data.

## 4. Select Epidemiological Studies Linking Phthalate Exposure and Health Outcomes

Controlled laboratory animal studies on the toxic effects of phthalates have enabled understanding of biological plausibility and potential mechanisms of actions of this class of chemicals. Thus far, the majority of the laboratory animal exposure/toxicity studies have focused on DEHP and DBP/DiBP, with limited studies examining the toxicities of other phthalates [164,165,166,167,168,169,170,171,172,173,174,175,176,177,178,179,180,181,182,183,184,185,186,187,188,189,190,191]. The reproductive and developmental effects of phthalates are among the most studied and well-described toxic endpoints in those studies. The toxic endpoints determined in animal studies, following phthalate exposure, include retention of nipples, anogenital distance, pathological changes in testes and male reproductive accessory glands, hypospadias, cryptorchidism, and semen parameters. Phthalates have well-documented anti-androgenic activity in rodent studies that result in reduced circulating testosterone. Several reviews have been published on the toxicity of phthalates [10,14,168,169,170]. As a class of well-studied endocrine disrupting chemicals, exposure to phthalates has been linked to sex anomalies, endometriosis, altered reproductive development, early puberty and fertility, breast and skin cancer, allergy and asthma, overweight and obesity, insulin resistance, and type II diabetes.

### 4.1. Diabetes

Diabetes is a metabolic disease that results in elevated blood glucose levels. Epidemiological studies in the US [192,193] reported that women with higher urinary concentrations of MBP, MiBP, MBzP, and MCPP and those of DEHP metabolites showed increased risk of diagnosis for diabetes in comparison with those who had lower concentrations of phthalates. Phthalate exposures have been shown to result in insulin resistance [166,194].

### 4.2. Overweight and Obesity

Overweight and obesity can be associated with many chronic diseases, including diabetes. Phthalate exposure was associated with increased body mass and waist circumference [195]. Some phthalate metabolites (MEP, MBP, and MiBP) were associated with obesity in children, whereas MEHP, MECPP, MEHHP, MEOHP, MBzP, and MCNP were associated with obesity in adults. Further, DEHP metabolites were found to be significantly associated with obesity in adult females and older males [196]. Urinary concentration of MBP was associated with fat deposition in boys in China [197].

Several studies have shown a significant association between obesity and phthalate exposure [193,196,198]. MEP, MEHP, MBzP, MEHHP, and MEOHP were associated with obesity in the US population [198]. MBzP, MEHHP, MEOHP, and MEP were associated with increased waist circumference and BMI [193] In contrast, higher concentrations of MEP and DEHP were found in the serum and urine of individuals who were undergoing weight loss [199]. Food intake is the main source of phthalate exposure (for high molecular weight phthalates). Therefore, overweight population with high food intake might have high phthalate exposures.

### 4.3. Allergy and Asthma

Exposure to high molecular weight phthalates are is associated with allergies and asthma [200,201]. Studies indicated that children are prone to exposure to DEHP, BzBP, DBP, and DEP and that exposure was associated with allergic rhinitis, atopic dermatitis, and conjunctivitis [202]. DEHP and BzBP and their monoesters are regarded as allergens, and exposure to them has been associated with asthma and wheezing in adults [200,201]. Exposure of DEHP, BzBP, DBP, and DEP during gestation has been associated with allergic responses in infants and toddlers [200]. Urinary MEHP concentrations are correlated with asthma in children [203]. Prenatal exposure to DEHP metabolites and BzBP has been associated with the risk of developing asthma at the age of 7 years and older [204].

### 4.4. Reproductive Health

Urinary MEP and MBP and the metabolites of DEHP and DiNP are associated with anomalies in pubertal development in girls [205]. A significant association between urinary concentrations of MBzP, MEHP, and MEP and increased risk of endometriosis was found in women [206]. Exposure to MEP, MiBP, and MBP pose an increased risk of pregnancy loss in Chinese women.

Poor semen quality was associated with exposure to phthalate metabolites. MBP and MBzP were strongly associated with spermatotoxicity and subfertility in males [32,54]. Significantly higher concentrations of DEHP (4.66 µg/mL) and MEHP (3.19 µg/mL) were found in the urine of 40 Turkish boys with gynecomastia as compared to that of control groups [207]. Several reviews have appeared on the reproductive and developmental toxicities of phthalates [208]. Whereas some inconsistencies exist across phthalates for specific health outcomes associated with exposures, moderate to strong evidence of male reproductive effects have been demonstrated in the literature [208]. Because humans are exposed to thousands of harmful chemicals, establishing the link between exposure to a single substance class and adverse health outcomes is fraught with uncertainties.

## 5. Conclusions and Perspectives

Human biomonitoring studies are useful in elucidating exposures and body burdens of phthalates at a population level. Although the sources of exposure to phthalates are well described, several questions about cumulative exposures to phthalates throughout the life span, relative contributions of various sources to cumulative exposures, and mixed exposures that may include phthalates or other chemicals that may elicit common adverse outcomes remain unanswered. Biomonitoring studies clearly demonstrate that human exposures are almost ubiquitous, and, in most cases, children have higher exposures than do adults. The existing studies indicate that the observed associations between phthalate exposure and disease outcomes are exploratory and preliminary, the health effects of phthalate exposure warrant further study. Robust analytical methods exist to measure more than 20 phthalate metabolites in urine, a preferred matrix of choice for biomonitoring studies. Although studies have reported the occurrence of phthalate metabolites in other human specimens, including serum, seminal plasma, and amniotic fluid, the relevance of these matrices in understanding toxic effects needs further investigation. Although biomonitoring studies select major biomarkers/metabolites of phthalates, several other intermediate and transformation products of phthalates appear to exist in human specimens. These intermediates may have more pronounced effects on health. Lack of analytical standards hinders the identification of those intermediate biological transformation products of phthalates. Further, the interaction of phthalate metabolites with other contaminants should be considered in future investigations.

There is a lack of biomonitoring data on phthalate exposures in developing countries in Africa and South America. Studies are needed in those regions with regard to exposures and associated health outcomes in populations. Further, epigenetic effects of phthalate exposures warrant further investigation.

## Figures and Tables

**Table 1:** Major phthalate diesters and their corresponding metabolites studied in the literature.

Parent Compounds	Abb.	Major Metabolites	Abb.
Dimethyl phthalate	DMP	Mono-methyl phthalate	MMP
Diethyl phthalate	DEP	Mono-ethyl phthalate	MEP
Dibutyl phthalate	DBP	Mono-n-butyl phthalate	MBP
Benzylbutyl phthalate	BzBP	Mono-benzyl phthalate (some mono-n-butyl phthalate)	MBzP
Dicyclohexyl phthalate	DCHP	Mono-cyclohexyl phthalate	MCHP
Di-2-ethylhexyl phthalate	DEHP	Mono-2-ethylhexyl phthalate	MEHP
		Mono-(2-ethyl-5-hydroxyhexyl) phthalate	MEHHP (5OH-MEHP)
		Mono-(2-ethyl-5-oxohexyl) phthalate	MEOHP (5oxo-MEHP)
		Mono-(2-ethyl-5-carboxypentyl) phthalate	MECPP (5cx-MEPP)
		Mono-(2-carboxymethyl-hexyl) phthalate	MCMHP (2cx-MMHP)
Diisobutyl phthalate	DiBP	Mono-isobutyl phthalate	MiBP
Diisononyl phthalate	DiNP	Mono-isononyl phthalate	MiNP
		Mono-(carboxyisooctyl) phthalate	MCiOP
Diisodecyl phthalate	DiDP	Mono-(carboxynonyl) phthalate	MCNP
		Mono-(carboxyisononyl) phthalate	MCiNP
Di-n-hexyl phthalate	DnHP	Mono-n-hexyl phthalate	MHxP
Di-*n*-octyl phthalate	DnOP	Mono-n-octyl phthalate	MnOP
		Mono-(3-carboxypropyl) phthalate	MCPP
		Mono-carboxy-n-heptyl phthalate	MCHpP
		Mono-n-heptyl phthalate	MHpP
		Mono-n-pentyl phthalate	MPeP
		Mono-iso-propyl phthalate	MiPrP

**Table 2:** Human exposure doses to total phthalates for the US population through various pathways (µg/kg bw/d).

Exposure Route	Dust Ingestion	Dust Dermal Absorption	Personal Care Products (Dermal)	Diet	Indoor Air Inhalation
Infants (<1 y *)	1.12	0.001	0.0095	-	0.845
Toddlers (1–3 y)	1.7	0.0008	0.0059	-	0.423
Children (3–11 y)	0.468	0.0006	-	4.68	0.203
Teenagers (11–18 y)	0.291	0.0005	-	-	0.089
Adults (>18 y)	0.233	0.0002	0.013–0.49	1.03	0.07

* y = years old; “-” means not reported; data source: Tran and Kannan, 2015 [46].

**Table 3:** Reported concentrations of major phthalate metabolites in human specimens collected from various countries.

Matrix	Country/Region	Studied Population	Concentration						Reference
			MMP	MEP	MBP	MiBP	MDEHP	Unit	
Urine	Australia	30 non-occupational exposure		18.5	11.8	7.3	25.2	μg/L; median	[61]
Urine	Austria	251 children/adolescents; 272 adults; 72 senior citizens		25	10	28	15.5	μg/L; median	[62]
Urine	Belgium	261 persons		34.3	33.3	24.3	11.7	μg/L; median	[63]
Urine	Belgium	210 adolescents			38.5		52.7	μg/L; median	[64]
Urine	Belgium	123 men 138 women		37.6	31.3	26.2	17.1	μg/L; median	[65]
Urine	Belgium	25 persons		20.4	15.6	15.9	12.01	μg/L; median	[66]
Urine	Brazil	300 children (6–14 years old).	8.3	57.3	42.4	43.8	109	μg/L; median	[67]
Urine	Canada	3236 persons (6–49 years old)		49.1	23.8		40.9	μg/L; median	[68]
Urine	Canada	2000 women (first trimester)		32.02	11.59			μg/L; GM	[69]
Urine	Canada	80 infants				7.01	10.63	μg/L; median	[70]
Urine	China	108 young adults	31.8	37.5	67	57.2	65.3	μg/L; median	[71]
Urine	China	21 women 19 men	16.5	20.7	49.6	44	44.2	μg/L; median	[72]
Urine	China	430 children (208 girls and 222 boys)	15.7	4.14	21.9		14.3	μg/L; median	[73]
Urine	China	183 samples	14.6	22.1	63.5	57.1	76.1	μg/L; median	[51]
Urine	China	364 males (19–44 years old)		28.2	47.1		42	μg/L; median	[74]
Urine	China	39 children (5–9 years)		28.5	232	81.3	79.1	μg/L; median	[75]
Urine	Czech	117 women	ND	56.7			32.2	μg/L; median	[76]
Urine	Czech	120 children	ND	31.6			61.9	μg/L; median	[76]
Urine	Denmark	60 men		54.5	36.8	47.3	68.1	μg/L; median	[77]
Urine	Denmark	145 women		74	26	48	67	μg/L; GM	[78]
Urine	Denmark	143 children		28	39	74	99	μg/L; GM	[78]
Urine	Denmark	129 children		29	111		107	μg/L; median	[79]
Urine	Denmark	441 children		16.6	80.1	72.2	89.8	μg/L; median	[80]
Urine	Europe	171 individuals		49.9	0		4.5	µg/g CR; median	[42]
Urine	Europe	1335 children		34.4	38.4	45.4	47.6	μg/L; median	[81]
Urine	Europe	1347 mother		48.2	23.9	30.1	29.2	μg/L; median	[81]
Urine	Europe	1301 mother		72	18.3	23.3	22.4	μg/L; median	[82]
Urine	France	279 mothers		43.5	35.7	53.7	84.6	μg/L; median	[83]
Urine	Germany	634 individuals			109	35.4	45.3	μg/L; median	[84]
Urine	Germany	254 children					99.9	μg/L; median	[85]
Urine	Germany	53 women 32 men		90.2	181		83.3	μg/L; median	[86]
Urine	Germany	399 individuals			49.6	44.9	38.8	μg/L; median	[87]
Urine	Germany	120 females and 120 males			19.6	25.5	19.3	μg/L; median	[41]
Urine	Germany	30 males and 30 females (2015)	2.8	13.5	8.0	9.8	12.3	μg/L; median	[88]
Urine	Germany	30 males and 30 females (2007)	8.0	53.6	16.4	19.3	33.4	μg/L; median	[88]
Urine	Germany	111 children (48 girls and 63 boys)			53.6	74.9	130.1	μg/L; median	[89]
Urine	Germany	465 children (8–10 years old)			52.5	62.8	75.7	μg/L; median	[90]
Urine	Germany	599 children			95.6	94.3	174.6	μg/L; median	[91]
Urine	Germany	600 children (3–14 years old)			96		85	μg/L; median	[91]
Urine	Germany	207 infants (1–5 month)		12.1			1.1	μg/L; median	[92]
Urine	Germany	104 mothers		50.5		66.6	28.9	μg/L; median	[93]
Urine	Germany	104 children		39.1	56.5	103.9	55.7	μg/L; median	[93]
Urine	Greece	239 women		142	32.1	36.7	44.6	μg/L; median	[94]
Urine	Greece	239 children		35.3	23.3	36	45.6	μg/L; median	[94]
Urine	Hungary	115 women	ND	55			32.4	μg/L; median	[76]
Urine	Hungary	117 children	ND	47			56.7	μg/L; median	[76]
Urine	India	15 women 7 men	8.6	150	13	18.3	77.9	μg/L; median	[72]
Urine	Iran	56 children and adolescent (6–18 years)	17.4	28.2	42.9		44.9	μg/L; median	[95]
Urine	Ireland	120 mothers		50.2	18.5	23.8	17	μg/g CR; GM	[96]
Urine	Ireland	120 children		38.7	26.1	41.4	32.8	μg/g CR; GM	[96]
Urine	Israel	205 adults (20–74 years old)			27.9	37.6	81.7	μg/L; median	[97]
Urine	Italy	83 women (2011)		73.1	38.8		15.6	μg/g CR; median	[42]
Urine	Italy	111 women (2016)		49.9	0		4.5	μg/g CR; median	[42]
Urine	Italy	83 females		61.0	32.5		10.5	μg/L; median	[98]
Urine	Italy	74 males		73.2	41.2		15.2	μg/L; median	[98]
Urine	Japan	8 women 27 men	18.2	16.4	17.7	7.5	35.1	μg/L; median	[72]
Urine	Japan	80 women (controls)		21.4	84.3		72.7	μg/L; median	[99]
Urine	Japan	57 women (cases)		39.6	87.2		89.3	μg/L; median	[99]
Urine	Japan	35 adults 1 children	33	18	36		5	μg/L; median	[100]
Urine	Japan	111 pregnant women	5.70	7.75	46.6		18.5	μg/L; median	[101]
Urine	Korea	39 children		19.2	107	53.4	145.6	μg/L; median	[102]
Urine	Korea	60 individuals	10	13.4	16.7	4.5	43.6	μg/L; median	[72]
Urine	Korea	25 adults		80	134	40.4	125.8	μg/L; median	[103]
Urine	Korea	305 women			41		23.7	μg/g CR; median	[104]
Urine	Korea	1646 elderly people			39.5		44.8	μg/L; median	[105]
Urine	Korea	6478 adults			44.2		88.2	μg/L; median	[106]
Urine	Korea	6003 adults			24.2		52.2	μg/L; median	[107]
Urine	Korea	171 children		2.71	12.4	5.25	12.3	μg/L; median	[108]
Urine	Korea	392 children					185	μg/L; median	[109]
Urine	Korea	265 mothers					67.4	μg/L; median	[109]
Urine	Korea	297 adults					55.7	μg/L; median	[109]
Urine	Kuwait	22 women 24 men	10.1	411	113	54.1	180.4	μg/L; median	[72]
Urine	Malaysia	19 women 10 men	6.3	18.6	10.5	10.8	27.5	μg/L; median	[72]
Urine	Netherlands	100 women	ND	112	43.2	41.3	61.8	μg/L; median	[110]
Urine	Norway	10 women	2	310	41.1	57	112.3	μg/L; median	[111]
Urine	Norway	61 adults		24.2	13.4	12.8		μg/L; median	[112]
Urine	Norway	116 pregnant women		55	25	20	26	μg/L; median	[113]
Urine	Portugal	112 children (4–18 years)		59.4	12.7	16.9	40.4	μg/L; median	[114]
Urine	Saudi Arabia	130 individuals	8.65	47.5	38.5	38.5	117.1	μg/L; median	[26]
Urine	Slovakia	129 occupational exposure			110	39.2	55.9	μg/L; median	[115]
Urine	Slovakia	68 occupational exposure population		201	103	61.4	82.7	μg/L; median	[116]
Urine	Slovakia	125 women	ND	54.8			36.7	μg/L; median	[76]
Urine	Slovakia	127 children	ND	39.6			82.8	μg/L; median	[76]
Urine	Slovakia	85 occupational exposure		78.5	85.6		21.5	μg/L; median	[117]
Urine	Slovakia	70 general population		78.1	96		14.7	μg/L; median	[117]
Urine	Spain	391 pregnant women		246	27.1	28.4	87.8	μg/L; median	[118]
Urine	Spain	120 children		198.9			63	μg/g CR; GM	[119]
Urine	Spain	120 mothers		150.8			33.3	μg/g CR; GM	[119]
Urine	Sweden	314 men		41	47		48.4	μg/L; median	[120]
Urine	Sweden	38 women	1.2	35	46	16	35	μg/L; median	[24]
Urine	Taiwan	41 women and 19 men (21–67 years)	32.3		36.5		15.9	μg/L; median	[121]
Urine	Taiwan	155 women	5.7	25.3	80		22.6	μg/L; median	
Urine	Taiwan	30 (children, age: 2)			100.4	17.2	195.8	μg/L; median	
Urine	Taiwan	59 (children, age: 5)			75.2	25.2	148.9	μg/L; median	
Urine	Taiwan	100 women			72.3	12.5	96.8	μg/L; median	
Urine	U.S.	45 males (subfertile couples)		108	24.7		91.4	μg/L; median	[122]
Urine	U.S.	35 children		177.7	52.4	16.6	1025.9	μg/L; median	[123]
Urine	U.S.	7600–10,031 individuals	1.4	167	18.9	3.6	73.1	μg/g CR; median	[124]
Urine	U.S.	12–18 months toddlers		13.2-1388	6.6–2540		<1.7–47.3	μg/L; median	[125]
Urine	U.S.	186 persons in Northern Manhattan		199	36			μg/L; median	[126]
Urine	U.S.	446 pregnant women		41.1				µg/g CR; GM	[127]
Urine	U.S.	378 pregnant women		47	13.7	9.47	14	μg/L; median	[128]
Urine	U.S.	482 individuals		141	17.8	7.6	106.6	μg/L; median	[27]
Urine	U.S.	2772 adults		167			35.4	μg/g CR; median	[129]
Urine	U.S.	392 children of 6–11 years old		96.9			69.9	μg/g CR; median	[129]
Urine	U.S.	2350 individuals	1.8	194.4	20.7	3.7	73	μg/L; median	[130]
Urine	U.S.	33 lactating women					35.7–45.9	μg/L; median	[131]
Urine	U.S.	50 pregnant women (18–38)		61.5	18.2		31.1	μg/L; median	[132]
Urine	U.S.	406 men	4.5	145	14.5		5.2	μg/L; median	[133]
Urine	Vietnam	16 women 14 men	8.4	7.2	19.1	13.6	56.7	μg/L; median	[72]
Serum	Denmark	60 men		<LOD	ND	<LOD	8.4	μg/L; median	[77]
Serum	Sweden	36 women		0.5	0.5	0.5	0.5	μg/L; median	[24]
Seminal plasma	Denmark	60 men		<LOD	<LOD	ND	<LOD	μg/L; median	[77]
Breast milk	Denmark	65 women	0.1	0.9	4.3		9.5	μg/L; median	[25]
Breast milk	Finland	65 women	0.1	1.0	12.0		13.0	μg/L; median	[25]
Breast milk	Sweden	42 women		ND	0.5	ND	0.49)	μg/L; median	[24]
Milk	Switzerland	54 women			6.0	24.3	26.2	μg/L; median	[134]
Milk	U.S.	33 lactating women					0.3–0.7	μg/L; median	[131]
Nail	Belgian	10 individuals		64	74		138	µg/g CR; median	[135]
Nail	Norway	61 adults	89.7	104.8	89.3			µg/g CR; GM	[112]

MDEHP: Sum of five DEHP metabolites (MEHP, MEHHP, MEOHP, MECPP, and MCMHP); ND: Not detected; LOD: Limit of detection; LOQ: Limit of quantification; GM = Geometric mean; CR = Creatinine.
